# Advances in Wearable Biosensors for Wound Healing and Infection Monitoring

**DOI:** 10.3390/bios15030139

**Published:** 2025-02-23

**Authors:** Dang-Khoa Vo, Kieu The Loan Trinh

**Affiliations:** 1 College of Pharmacy, Gachon University, 191 Hambakmoe-ro, Yeonsu-gu, Incheon 21936, Republic of Korea; 2 BioNano Applications Research Center, Gachon University, 1342 Seongnam-daero, Sujeong-gu, Seongnam-si 13120, Republic of Korea

**Keywords:** wearable biosensors, wound healing, infection monitoring, real-time monitoring, smart healthcare devices

## Abstract

Wound healing is a complicated biological process that is important for restoring tissue integrity and function after injury. Infection, usually due to bacterial colonization, significantly complicates this process by hindering the course of healing and enhancing the chances of systemic complications. Recent advances in wearable biosensors have transformed wound care by making real-time monitoring of biomarkers such as pH, temperature, moisture, and infection-related metabolites like trimethylamine and uric acid. This review focuses on recent advances in biosensor technologies designed for wound management. Novel sensor architectures, such as flexible and stretchable electronics, colorimetric patches, and electrochemical platforms, enable the non-invasive detection of changes associated with wounds with high specificity and sensitivity. These are increasingly combined with AI and analytics based on smartphones that can enable timely and personalized interventions. Examples are the PETAL patch sensor that applies multiple sensing mechanisms for wide-ranging views on wound status and closed-loop systems that connect biosensors to therapeutic devices to automate infection control. Additionally, self-powered biosensors that tap into body heat or energy from the biofluids themselves avoid any external batteries and are thus more effective in field use or with limited resources. Internet of Things connectivity allows further support for remote sharing and monitoring of data, thus supporting telemedicine applications. Although wearable biosensors have developed relatively rapidly and their prospects continue to expand, regular clinical application is stalled by significant challenges such as regulatory, cost, patient compliance, and technical problems related to sensor accuracy, biofouling, and power, among others, that need to be addressed by innovative solutions. The goal of this review is to synthesize current trends, challenges, and future directions in wound healing and infection monitoring, with emphasis on the potential for wearable biosensors to improve patient outcomes and reduce healthcare burdens. These innovations are leading the way toward next-generation wound care by bridging advanced materials science, biotechnology, and digital health.

## 1. Introduction

Wound healing is a life-supporting process involving the restoration of tissue function and the integrity of the body. It is an intricately complex process that is chronological but partly overlapping through classic phases of hemostasis, inflammation, proliferation, and remodeling [1,2]. Although small-sized wounds can be intrinsically repaired by the human body, large-sized or complex wounds, such as chronic wounds, diabetic ulcers, or surgical incisions, pose immense challenges toward their effective healing [3,4,5]. Among these barriers, infection is recognized as one of the critical determinants in delayed wound healing and a burden to healthcare [6,7]. Infections amplify inflammation, retard tissue repair, and increase the possibility of systemic complications [8]. Adding to this, the worldwide spread of antimicrobial resistance has further complicated and increased the costs of managing wound infections [9]. The effective evaluation of the condition of wounds is of paramount importance for the early detection of complications and intervention [10]. However, traditional methods of wound care are usually based on subjective clinical assessment and discrete laboratory tests, which often result in delayed interventions with less-than-optimal outcomes. Such limitations show why new solutions are needed that can provide continuous real-time data regarding wound health.

Wearable biosensors have developed into a new tool for tackling various wound management-related issues [11]. As wearable sensors allow for continued monitoring of key parameters like pH, temperature, moisture levels, and inflammatory markers in wound care, such sensor integration brings accuracy and promptness that had never been imagined in the treatment of a wound [12,13]. They are also capable of detecting slight changes in the wound milieu, which might signal an infection or the impairment of the healing process, respectively, enabling early intervention and minimizing chances of complications [14]. Materials and integrated technologies of the current generation of wearable biosensors now have a substrate that provides flexibility, a microfluidic system, and wireless modules for communication [15]. This facilitates easy integration with patient physiology and enables remote functions for monitoring. Integration of artificial intelligence and data analytics further enhances the understanding of complex biomarker data, thus helping in predictive diagnostics and personalized treatment strategies [16]. Wearable biosensors represent a critical shift toward proactive and efficient wound management by connecting conventional wound care practices with modern digital health technologies [17].

This review will discuss the state-of-the-art technologies and applications of wearable biosensors for wound healing and infection monitoring. It points out the leading-edge technologies in sensor design, material science, and data integration that have driven development. This review also discusses the tremendous potential of wearable biosensors to revolutionize wound care by improving diagnostic accuracy, allowing real-time therapeutic interventions, and improving patients’ general outcomes. It also details the main difficulties and limitations the technology needs to overcome before being widely accepted and adopted in clinics, relating to its regulation, cost, patient compliance, and finally, technical constraints. Sensor accuracy is of paramount importance for the dependable detection of biomarkers since even slight deviations can affect clinical decision making. Biofouling, resulting from the adsorption of biological material on sensor surfaces, can also deteriorate performance over time and, consequently, result in the need for novel surface modifications to restore function. Power constraints present significant challenges as well, especially for continuous long-term monitoring, and therefore the necessity for low-energy, efficient designs or self-powered devices. Addressing these issues is important for the facile integration of wearable biosensors in clinical routines. That is why the review provided, by taking an overview of state-of-the-art wearable biosensors, gives directions for further research and encourages novel solutions for changing the standards of worldwide wound care.

## 2. Biological Basis of Wound Healing and Infection

### 2.1. Stages of Wound Healing

**Figure 1:**
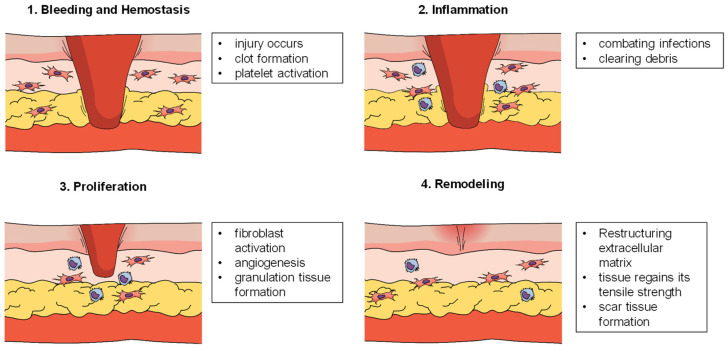
Stages of wound healing. Created with mindthegraph.com.

The four interrelated phases of wound healing—hemostasis, inflammation, proliferation, and remodeling—are part of a complicated, multifaceted biologic process [18,19]. Many internal and external factors influence the course of these phases, which are all crucial and interconnected in restoring the integrity and function of damaged tissue [20,21] (Figure 1).

The first stage, hemostasis, immediately follows injury and serves to arrest bleeding and establish a provisional matrix for later cellular activities [22,23]. After the compromise of tissue integrity, aggregated platelets undergo activation that leads to the formation of a clot by the activation of the coagulation cascade. The formed clot not only stemmed further loss of blood but also released growth factors such as platelet-derived growth factor (PDGF) and transforming growth factor-beta (TGF-β) that, in later steps, recruit immune cells and fibroblasts to the wound bed. A fibrin mesh formed during hemostasis provides a structural scaffold for cell migration in later steps. In this phase, biomarkers such as PDGF and TGF-β exhibit an early increase. These molecules play essential roles in clotting to stop bleeding and form a provisional matrix for healing. They rise sharply and then drop as the clot becomes stable [24].

The inflammatory stage follows hemostasis and usually lasts from 24 to 48 h, although it can go on for longer in chronic wounds [22,25]. During this stage, neutrophils and macrophages invade the wound site and play a dual role: the clearance of pathogens and dead cells, and the release of cytokines such as interleukin-1 (IL-1) and tumor necrosis factor-alpha (TNF-α). These inflammatory mediators increase further the recruitment of immune cells, orchestrating the switch into the proliferative phase. Though an inflammatory response is important to decontaminate the wound, too much or excess inflammation delays the healing and may cause the formation of non-healing chronic wounds. Biomarkers like IL-1 and TNF-α increase because of the migration of immune cells. Although elevated early during this phase, they fall progressively as the inflammation resolves [24].

The proliferative phase is when the repair process is shifted from immune defense to tissue renewal [26,27]. The latter, occurring under angiogenesis and the proliferation of fibroblasts during this phase dominated by both, synthesizes collagen and the other components of the extracellular matrix to finally form a granulation tissue serving as a scaffold for newly formed blood vessels. Endothelial cells proliferate and form capillary networks for the supply of oxygen and nutrients to the wound site. Keratinocytes also migrate across the wound bed to contribute to re-epithelialization and restoration of the skin barrier. The process starts with the production of matrix metalloproteinases (MMPs) by fibroblast, which facilitates fibroblast movement within the matrix; therefore, this biomarker is predominant in this phase. This process is tightly regulated by transforming growth factor β (TGF-β) [24].

The last is the phase of remodeling or maturation: strengthening and restructuring of newly formed tissue; this might take some months to years, given the size of the wound and type [28]. Collagen type III which was laid down during the proliferation gets replaced with collagen type I, increasing tensile strength. Wound contraction is mediated by myofibroblasts and, thereby, its size gets reduced. The extracellular matrix reorganizes while capillaries formed in the proliferative phase slowly start regressing, therefore leaving a more stabilized and functioning tissue. Inflammatory cytokines (e.g., IL-1) return to normal, indicating resolution. Enzymes such as MMPs control the remodeling of the extracellular matrix, and these are reduced as the wound closes [24].

**Figure 2:**
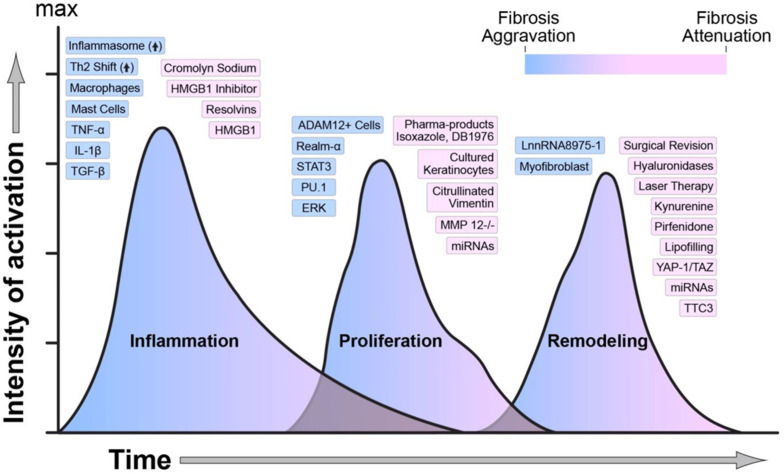
Regulators of wound healing and fibrosis. The timing, overlap, and intensity of activation in each phase of wound healing are governed by several molecular, biological, and mechanical variables. The image illustrates how each of these elements influences wound healing. Blue indicates activation, while pink denotes attenuation of fibrosis. Copyright MDPI (2020) [29].

These phases are important to understand because they provide critical knowledge on how disruptions in infections, comorbidities, or a lack of proper care can delay the process and impair wound healing. These also help inform the development of therapeutic approaches, including wearable biosensors applied at each step in the process of wound healing. Figure 2 [29] provides an overview of the molecular mediators and key biomarkers that regulate each phase of wound healing, offering a foundational understanding of their dynamic roles.

### 2.2. Role of Biomarkers in Assessing Wound Status and Infections

Biomarkers may be important in assessing wound status and detecting infection because they will provide real-time information concerning the wound-healing process and the possible development of complications [30,31]. These biomarkers, being physically, chemically, and biochemically observable, are, therefore, very important in monitoring the conditions within wounds so that any appropriate interventions may be promptly provided at a clinical level.

pH is one of the important biomarkers in wound assessment because it will have a direct effect on enzyme activity, microbial growth, and cellular processes involved in the healing process [32]. Healthy skin has a slightly acidic pH, ranging from 4.5 to 6, which provides an unfavorable environment for pathogenic bacteria [33]. In contrast, chronic wounds usually have an elevated pH of 7–9, which may promote microbial proliferation and impair healing [34]. The continuous monitoring of pH may therefore give important feedback about wound progress and therapeutic intervention efficacy [35].

Cytokines, such as interleukins (e.g., IL-6, IL-8) and TNF-α, are critical inflammatory mediators that reflect the immune response within a wound [36]. High levels of pro-inflammatory cytokines usually signal infection or chronic inflammation, while a balanced cytokine profile is necessary for the transition into the proliferative phase of healing [37]. Assessment of cytokine levels aids in the differentiation between normal and pathological inflammation, providing information about the status of the wound [38].

Temperature variations seen in a wound may point to either inflammation or infection [39,40]. For example, localized temperature rise may provide an indication of active immune responses or the presence of colonization by bacteria; abnormal cooling may point to poor blood flow or ischemia [41]. Integrating infrared sensors with thermal imaging techniques into wearable biosensors gives non-invasive modes of temperature monitoring [42,43]. Reactive oxygen species (ROS), including superoxide and hydrogen peroxide, are byproducts of cellular metabolism that play a dual role in wound healing [44]. While low levels of ROS are involved in cell signaling and antimicrobial defense, excessive ROS can damage tissues, delay healing, and exacerbate chronic wounds [45,46]. ROS level-detecting biosensors may, therefore, help in the assessment of oxidative stress and guide antioxidant therapies [47,48].

Infection-related metabolites like trimethylamine (TMA) and uric acid (UA) have been increasingly recognized as very important biomarkers that are used in the identification and monitoring of wound infections [49,50]. TMA is usually produced as a volatile organic compound from bacterial metabolism; thus, it is mainly related to anaerobic bacterial activity [51]. It may signal bacterial colonization or biofilm formation in wounds, particularly those involving chronic infections. Moreover, the detection of TMA using wearable biosensors will provide rapid and non-invasive diagnosis, and clinicians can therefore intervene promptly to prevent infection progression [52]. UA, a byproduct of purine metabolism, seems to play a dual role in wound healing and infection monitoring [52]. High levels of UA in wound exudates are often associated with oxidative stress and inflammation—common hallmarks of infected or poorly healing wounds [53]. High UA levels can also enhance inflammation through the promotion of ROS formation and the activation of an immune response. Tracking UA in wound fluids might give insight into the inflammatory status and metabolic environment involved, enabling a better understanding of wound healing dynamics [54].

Other markers also exist: glucose, lactate, and bacterial metabolites contribute more depth to the picture of wound health [7,55]. In individuals with diabetes, high levels of glucose are present in the wounds themselves, leading to delayed wound healing [56,57]. Conversely, infection is generally accompanied by increasing numbers of bacterial metabolic products like ammonia [58].

Monitoring these biomarkers with wearable biosensors not only facilitates early detection of complications but also enables personalized wound care by tailoring interventions to the specific biochemical environment of the wound. This approach ultimately improves patient outcomes and reduces the burden of chronic wounds. Advanced biosensors that can detect such metabolites are becoming very instrumental in personalized wound care. The use of such tools will help guide therapeutic decisions to optimize treatments and reduce the chances of developing chronic infection complications, as it gives real-time insight into bacterial activity and metabolic imbalances—hence improving patient outcomes.

## 3. Advancements in Wearable Biosensor Technologies

### 3.1. Types of Sensors

Wearable biosensors form a diverse category, and different types are developed for targeting specific biomarkers [59,60]. Specifically, major classes of such devices include electrochemical, optical, and colorimetric sensors; all three have unique advantages and features with regard to wound care applications [61].

The dominance of electrochemical sensors is because they have very good sensitivity and specificity and are versatile, showing the ability to detect a wide range of biomarkers [62,63]. The working principle of such sensors is based on monitoring the electric signals that arise due to the interaction of a target analyte, such as glucose, pH, and cytokines, with a defined electrode [64]. Often, these electrochemical biosensors will be integrated into flexible, stretchable substrates so that they can be well applied in dynamic wound contexts. For example, pH-sensitive electrodes can monitor the acidity of a wound to provide continuous feedback on the progression of healing [65]. Their simplicity in fabrication and potential for miniaturization make them ideally suited for long-term monitoring in wearable formats. The choice of electrode material is central to establishing the sensitivity, stability, and selectivity of electrochemical biosensors [66]. The usual materials applied are carbon-based electrodes (graphene and carbon nanotubes), noble metals (gold and platinum), and conductive polymers. Carbon-based electrodes are widely utilized due to their improved conductivity, biocompatibility, and large surface area, whereas noble metals have improved electron transfer kinetics, thus improving detection limits [67]. Conductive polymers such as polyaniline or polypyrrole are very flexible and chemically tunable and therefore best suited for wearable biosensors [68]. Their associated modification methods also optimize sensor performance via enhanced surface reactivity and selectivity. Nanostructuring (e.g., nanoparticle coatings, nanowires), functionalization with biomolecules (e.g., enzymes, antibodies) [69,70], and chemical doping [71] are a few of them. Nanostructured surfaces enhance active sites for target interactions for improving sensitivity, and biomolecule functionalization for improving specificity for measuring biomarkers including glucose, lactate, or cytokines [72]. These factors all influence sensor performance via improved electron transfer efficiency, improved signal response, and long-term stable operation. Advanced electrode materials and modification methods facilitate the capability of electrochemical biosensors to attain rapid, precise, and real-time identification of wound biomarkers, thus being suitable for continuous infection monitoring and personal healthcare applications.

Optical sensors use the interaction between light and biological or chemical entities to allow the recognition of biomarkers; most of these systems use either fluorescence, absorbance, or reflectance measurements for specific analytes of interest in the wound environment [73,74]. Inflammatory biomarkers or ROS, for example, could be sensed by fluorescence-based sensors with high accuracy in order to reveal the inflammatory status of a wound [61,75]. Optical sensors also provide non-invasive measurements and can be integrated into patches or films placed onto the skin [76,77]. They, however, remain very sensitive and versatile, although complexity and higher cost than the electrochemical sensors could hamper wide-scale applications.

Colorimetric sensors are simple, cost-effective tools that visually indicate the presence or concentration of specific biomarkers through color changes [78,79]. These sensors are usually included in wound dressings or patches, in which the analytes react with, for example, pH, glucose, or bacterial metabolites [80]. Thus, an example of a pH-sensitive colorimetric patch is one that changes color from yellow to blue upon interaction with an alkaline environment of the wound, indicating possible infection [81,82]. The greatest advantage of colorimetric sensors lies in their application in point-of-care and at-home monitoring because they do not require any sophisticated instrumentation for data interpretation [83,84]. But it is their qualitative nature and lower sensitivity compared with other sensor types that may seriously restrict their application in complex clinical settings.

Improvements in sensor technologies, such as hybrid systems combining multiple detection methods, further continue to widen their applications in wound care [85]. Electrochemical sensors have been strong due to their precision and adaptability, optical sensors due to their advanced non-invasive abilities, and colorimetric sensors due to their simplicity and accessibility—all very important features in the different aspects of real-time wound monitoring.

### 3.2. Materials and Designs

The innovations in materials and design have transformed wearable biosensors for real-time wound monitoring into a device that is functional, comfortable, and adaptable. More precisely, the advances made in the area of flexible/stretchable materials, microneedles, and 3D-printed devices have significantly improved the performance of such systems while improving user-friendliness [86,87].

Modern wearable biosensors usually depend on flexible and stretchable materials as their substrate, enabling these devices to easily conform to the irregular shapes of human skin [88,89]. The more common materials include silicone elastomers, hydrogels, and conductive polymers, since they are compatible with biological systems and capable of bearing mechanical integrity for a longer duration of time [90,91,92]. These materials make the patient feel comfortable and simultaneously increase the ability of the sensor to keep constant contact with the wound site, which enhances accurate measurement. Subsequent years of improvement on nanomaterials increased the sensitivity and stability in flexible sensors using graphene and carbon nanotubes, respectively [93,94]. For example, graphene-based flexible electrodes sensed slight biochemical changes occurring in the wound site, thus assuring the detection of any form of complication at its earliest stage [95].

Microneedle-based biosensors represent a new, minimally invasive approach to wound monitoring [96]. These devices are composed of microscopic needles that penetrate only the upper layers of the skin to reach the interstitial fluid with no significant pain or harm caused. The microneedles can be made from biocompatible polymers, metals, or ceramics and are frequently integrated with electrochemical or optical sensing platforms [97]. They allow for the direct measurement of important biomarkers, such as glucose, cytokines, and lactate, by providing real-time data with high specificity. Apart from monitoring, the microneedles could also be combined with drug delivery systems to achieve closed-loop therapeutic interventions, once more offering a dual-function design for wound care [98,99].

3D printing has further transformed the designing and manufacturing of wearable biosensors [100,101]. It can be used to customize sensors using additive manufacturing techniques for a particular type of wound or anatomical location [102,103]. One could create a spatially defined architecture with 3D printing technology, like the placement of micro-channels for fluid sampling or intricate sensor arrays for the detection of multi-biomarkers. For example, the development of 3D-printed flexible patches with conductive inks for the monitoring of pH and temperature may provide a simultaneous, and therefore comprehensive, wound monitoring technique [104]. The method increases functionality in biosensors and enables fast prototyping and customization against individual patient needs.

Next-generation wearable biosensors with improved efficiency, being more patient-centric, and able to manage the complexity of wound healing and infection control are being made possible by such development of materials and designs.

### 3.3. Enhancing Technical Summaries with Key Performance Parameters

The incorporation of detection limits, sensitivity, and other performance factors is necessary to have a complete analysis of electrochemical biosensors [105]. These parameters give measurable information regarding the efficiency and credibility of the sensors in sensing wound biomarkers. For example, electrochemical pH sensors applied in wound monitoring have detection limits of as low as 0.01 pH units, allowing a wound’s risk of infection to be analyzed accurately [106]. In parallel, lactate biosensors, important for tissue oxygenation monitoring, have been developed to sensitivities of 2.9 µA/mM cm^2^, enabling real-time monitoring of ischemic states in wounds [107]. Advances in nanomaterial-modified electrodes in recent years have also boosted sensor performance. For instance, gold nanoparticle-modified electrodes facilitate electron transfer rates, amplifying signal response and lowering the limit of detection (LOD) to sub-micromolar levels for cytokines such as IL-6, a principal marker of inflammation [108]. Additionally, flexible substrate-based wearable electrochemical biosensors have exhibited high repeatability (>95%) and 60 days of stability for stable long-term monitoring [109]. The incorporation of these detailed performance data makes the discussions more accurate and effective at highlighting the implications of biosensors for early infection detection, real-time wound assessment, and improved patient management in clinical settings.

Table 1 presents clinical biomarkers including temperature, pH, and uric acid (UA), their normal and pathological concentration ranges, necessary detection sensitivities, and their particular roles in wound healing and infection quantitation.

Wound fluid is the ideal medium for the assessment of biomarkers, for example, pH, ROS, UA, and bacterial metabolites like ammonia (NH_3_), providing an immediate snapshot of the wound microenvironment and its progression, including infection, inflammation, and healing. Sensors to assess wound exudate are, however, susceptible to biofouling by the high protein content and cellular debris, potentially compromising sensor function [116]. On the other hand, capillary blood gives more reliable and stable measures of inflammatory biomarkers, e.g., cytokines IL-6 and TNF-α [117]. The medium is convenient for tracking systemic inflammatory responses and serves to supplement localized wound information. In addition, interstitial fluid plays a significant role in the monitoring of markers and metabolites in proximity to the wound, including glucose and lactate, that is being emitted from the wound bed, thereby offering further insight into tissue viability and cellular activity near the wound [118,119]. The sites of measurement highlight the complexity of the biological matrix of the wound, with varying susceptibility of the sites to biofouling. While interstitial fluid and capillary blood show less tendency for biofouling than wound fluid, they can pose additional complications such as sensor invasiveness or the requirement for integration with microneedles [120]. This finding highlights the need to take these factors into account when designing sensors, testing their performance, and planning their clinical use.

### 3.4. Integration with AI and IoT

The amalgamation of artificial intelligence and the Internet of Things in wearable biosensor systems has revolutionized wound monitoring and management, improving data analysis and remote care [112,121]. Synergy between these technologies has brought about smart, connected healthcare solutions that deliver actionable insights and better clinical outcomes.

AI is an integral part of processing and interpreting the vast amounts of complex data produced by wearable biosensors [122]. The usual methods of data analysis are not very good at defining subtle patterns or correlations in multidimensional datasets [123]. Machine learning algorithms, which are a subset of AI, do very well in that respect by identifying trends and anomalies in real time [124]. By means of example, AI algorithms might monitor pH, temperature, and cytokine level changes and predict infection before the actual clinical symptoms have had a chance to manifest [125]. Deep learning models in this space enhance this capability by combining multiple biomarkers into a holistic view of the wound environment, thus helping in timely interventions aimed at preventing complications and enhancing rates of healing [126]. The systems may also be designed with respect to individual patient profiles by AI to afford personalized treatment plans in accordance with specific conditions and healing trajectories.

IoT connectivity gives expanded capability to wearable biosensors in remote monitoring and sharing [127]. The information from the IoT-enabled sensors could be shared in real time through a secure, cloud-based platform, meaning continuous patient oversight without much ado about frequent clinical visits [128,129]. This is very helpful, especially for people residing in remote areas and far away from accessing conventional services for wound care [130]. The same IoT systems also provide access to wound data through mobile applications for both caregivers and patients, increasing engagement and adherence to care plans [131]. This integration of IoT with the telemedicine platform allows the deployment of collaborative care models where specialists can review the data and give recommendations—geographical constraints notwithstanding [132].

AI and IoT together also make it possible for closed-loop systems, in which biosensors track wound conditions and, using real-time data processing, initiate therapeutic reactions such as targeted drug administration [133]. To create a self-regulating care system, for example, a biosensor that detects elevated inflammatory markers may cause an automated release of anti-inflammatory drugs [134].

With continuous advancements in both AI and IoT, it is the integration of these two with wearable biosensors that has come into play for changing the dimensions of precision medicine in facilitating proactive, efficient, and patient-centered wound care solutions. In this way, improvement of healthcare access, efficiency, and responsiveness can be fully achieved.

## 4. Key Applications in Wound Healing and Infection Monitoring

### 4.1. Real-Time Monitoring

Advanced wound care based on real-time monitoring utilizes the concept of wearable biosensors continuously providing insight into the status of the wound environment [135]. Development is required in sensors that detect all major parameters, such as pH, temperature, and moisture, for assessment and prediction of wound status, further complications, and timely intervention guidance [136].

One of the most widespread applications of biosensors in wound care is pH monitoring [137,138]. A healthy wound environment usually has a slightly acidic pH which promotes tissue regeneration and prevents the colonization of pathogenic bacteria. On the other hand, an increase in pH to alkaline values usually signals either infection or a slowdown in healing. Sensors can be embedded with pH-sensitive materials, such as hydrogels or conductive polymers, to monitor such changes and report in real time [139,140]. For example, smart pH sensors integrated into the wound dressing could detect fluctuations in pH values without any need for intrusive sampling; clinicians could observe the status of wound healing. Another important application is the monitoring of temperature, which, through local wound temperature fluctuations, can indicate inflammatory responses or infections. Many wearable devices used for the tracking of temperature changes are based on infrared-based sensors or thermistor arrays. For example, a sustained temperature increase over the wound site may indicate bacterial colonization and, hence, early intervention [141]. Most temperature sensors are combined with multi-modal systems in order to give more holistic insights when integrated with other parameters such as pH or cytokine levels [142].

Moisture monitoring is a very critical aspect in the maintenance of optimal hydration levels necessary for wound healing. Excess moisture causes maceration and enhances bacterial growth, while dryness hampers cellular migration and the formation of granulation tissue [143]. Capacitive and resistive moisture sensors are in wide use for the measurement of water content within the wound environment. Most of the smart wound dressings include such sensors, which then alert caregivers or patients in case there is a need for change in dressing protocols [144].

Other examples of the advanced wearable device are the hydrogel-based patches for the monitoring of multiple parameters and colorimetric dressings, which provide visible changes in pH or moisture [112,145,146]. These systems can offer early detection of wound deviations from healthy status and thereby avoid possible complications of chronic infection or delayed healing.

**Figure 3:**
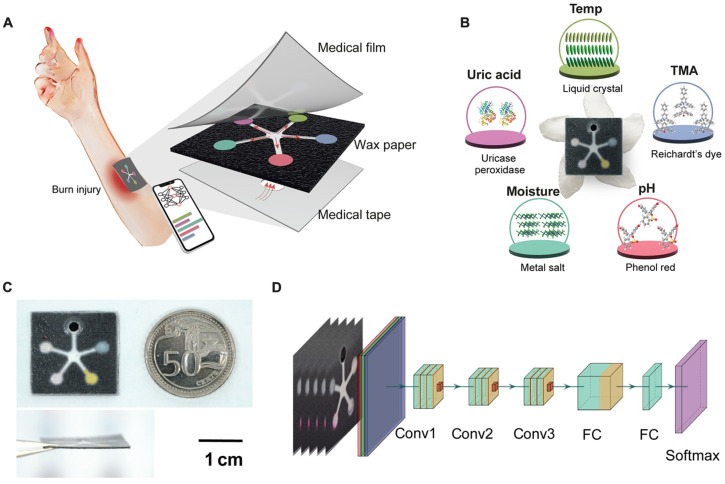
Schematic of the PETAL sensor, a wound healing monitoring sensor working without a battery. (**A**) A graphical abstract of the sensor adhered onto a burn wound for colorimetric analysis of wound healing progress. (**B**) The real sensor patch and the multiplexed sensing targets/principles. (**C**) The shape and dimension of the sensor patch compared to a 50 cent Singapore coin. (**D**) Schematic of neural network-based machine learning algorithm used for wound classification. Copyright AAAS (2023) [50].

The PETAL sensor patch is a new, paper-like, battery-free device designed for total wound care [50] (Figure 3). The name, PETAL, is apt because of its design, resembling a five-petaled pinwheel flower; each “petal” independently acts as a different sensing region.

The PETAL patch has been developed to facilitate real-time multi-analyte sensing through the detection of temperature, TMA, UA, moisture, and pH via individual sensing regions. There are colorimetric sensors in every sensing region that react based on the concentration of specific analytes. The sensor collects the wound exudate on a central opening and then distributes it to each sensing region. Thereafter, chemical reactions occurring in these regions result in color changes proportional to the concentration of specific biomarkers. The PETAL patch allows simultaneous sensing of all of the five wound markers mentioned above. As far as response times are concerned, the duration between a change in analyte concentration (for example, UA) and the visible color change is a function of the diffusion time of the analyte from the wound fluid to the sensor region. Typically, this delay is between 3 and 8 min, with variations possible depending on the specific analyte and the viscosity of the wound fluid. After sufficient wound fluid is accumulated (usually within a few hours or over a few days), the PETAL sensor patch will complete the detection of biomarkers within 15 min. The patch can also maintain continuous monitoring since color changes are visible as a result of changes in the concentration of analytes. Additionally, the sensor patch can operate without an energy source as sensor images are captured by a mobile phone and analyzed by AI algorithms to determine the patient’s healing status. The AI algorithm is capable of rapidly processing data from a digital image of the sensor patch for very accurate classification of healing status. This can be done without removing the sensor from the wound. In this way, doctors and patients can monitor wounds more regularly with little interruption to wound healing. Timely medical intervention can then be administered appropriately to prevent adverse complications and scarring. The operational lifetime of a PETAL patch depends on the wound environment conditions and exposure. Longer application can lead to biofouling or saturation of the sensing areas, which need to be replaced to maintain measurement accuracy. Calibration is an integral part of the PETAL patch design. Every sensor area is pre-calibrated during the manufacturing process with the use of standard solutions that have known concentrations of analytes. Calibration curves are established for every analyte in order to ensure an accurate interpretation of colorimetric changes. In addition, the patch has integrated calibration indicators that provide detection of sensor drift or fouling during its use, thus increasing its reliability.

Wound care is taking a proactive stance by using these sensors for real-time monitoring; this enables individualized treatment plans, which improve outcomes while decreasing healthcare expenditures.

### 4.2. Early Infection Detection

Early detection of infection in wounds is an important aspect in the prevention of complications and improvement of healing outcomes [147]. In this regard, wearable biosensors have become very important tools, targeting bacterial metabolites, enzymatic activity, and inflammation biomarkers to give real-time insight into the wound environment [148].

During microbial activity, bacterial metabolites, including ammonia, hydrogen sulfide, and short-chain fatty acids, are formed and may serve as indicators for the presence of pathogenic bacteria [149,150]. The biosensors used to detect such metabolites employ either chemical or electrochemical detection methods and could also be integrated into intelligent wound dressings. For example, sensors responding to the level of ammonia may track changes occurring in the microenvironment of a wound because an elevated level of ammonia is found with common bacterial infections [151]. The ammonia sensor is premised on the detection of the ammonia (NH_3_) gas or its ionic counterpart, ammonium (NH_4_^+^), as a key indicator of infection and bacterial presence in wound tissue [152,153]. The majority of the ammonia sensors integrated into wearable biosensors for wound monitoring are founded on electrochemical, optical, or colorimetric mechanisms in their detection schemes. Electrochemical sensors use modified electrodes with different materials like conducting polymers, metal oxides, or ion-selective membranes. The interaction of ammonia or ammonium with the electrode surface initiates a chemical reaction that produces a detectable alteration in electrical properties, including current, voltage, or impedance. For example, the rise in NH_3_ concentration in wound fluid changes the local pH, which can be detected using a pH-sensitive electrode [154,155]. These sensors are very specific and sensitive, and their response typically takes the order of seconds to minutes. Optical sensors make use of ammonia-sensitive dyes, where a change in ammonia concentration causes a shift in the absorption or fluorescence properties of the dye [156]. They are commonly incorporated into flexible substrates for real-time monitoring and are ideal for non-invasive applications since they create minimal disturbance to the wound bed. Colorimetric sensors rely on ammonia-sensitive chemicals incorporated into color-changing materials on exposure to NH_3_ [157]. The color is quantitatively proportional to ammonia concentration, and it is an inexpensive and simple way of detection. In wearable formats, these sensors are useful for point-of-care monitoring. Ammonia sensor performance relies on sensitivity, detection range, response time, and stability in complex wound exudates. In wound healing-based applications, such sensors are designed to sense NH_3_ concentrations in the micromolar to millimolar range because higher concentrations of ammonia are significantly correlated with infections and impaired healing processes. These devices are most useful in differentiating colonization from pathogenic biofilm formation, allowing timely intervention [158].

Another important indicator of infection is enzymatic activity, since some enzymes are released by bacteria or activated immune cells in response to infection [159]. Many sensors commonly monitor proteases such as matrix metalloproteinases—MMPs—and bacterial-specific enzymes such as lipases and hyaluronidases. High levels of MMPs, for example, are often seen in chronic wounds and tissue degradation, therefore biosensors for measuring MMPs is an emerging research field [160,161,162].

Biosensors containing enzyme-responsive elements, such as fluorogenic substrates or conductive polymers, can be developed to sense these enzymes, thus delivering quantitative information about the wound status [163]. The biggest advantage of these is specificity in defining different bacterial strains or the degree of inflammation.

Inflammation biomarkers, such as interleukins (e.g., IL-6, IL-8), C-reactive protein (CRP), and TNF-α, demonstrate the host’s immune response to the wound microenvironment. A large number of sensors that detect these biomarkers mainly rely on either immunoassay or electrochemical detection for accurate and highly sensitive detection. Wearable patches with built-in microfluidic channels are even able to collect the wound exudate and measure the level of cytokines in real time [164]. Persistent elevation of these biomarkers can indicate prolonged inflammation or infection, prompting necessary clinical actions.

**Figure 4:**
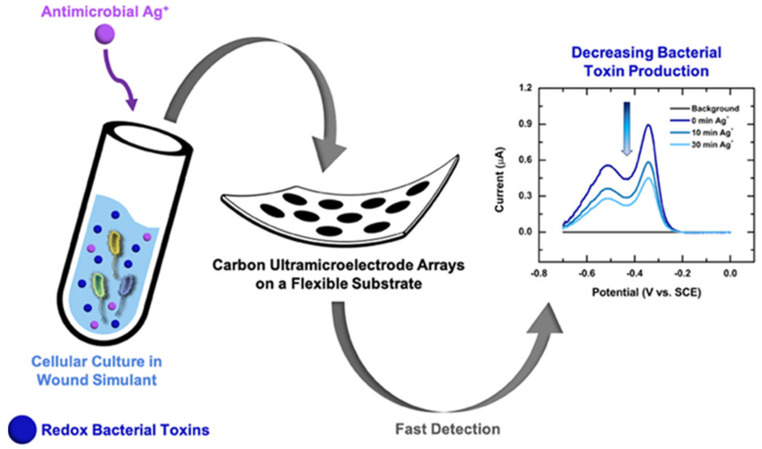
Fabrication and application of flexible carbon ultramicroelectrode arrays (CUAs) in electrochemical detection of multianalyte biomarkers for wound monitoring. Detection of pyocyanin (PYO), uric acid, and nitric oxide (NO•) as biomarkers in simulated wound media. Monitoring pathogen–host interactions and the effects of silver ions (Ag^+^) on PYO secretion by Pseudomonas aeruginosa. Quantification of cellular NO• from immune cells in the wound matrix using flexible CUAs. Copyright ACS Publications (2020) [165].

Various new sensors combine these methods to best monitor the wound in an all-inclusive manner: for example, hybrid sensors detecting bacterial metabolites coupled with inflammation markers offer multidimensional views of wound health [165] (Figure 4).

Such systems may even alert caregivers to the possibility of infection well before clear visible symptoms appear, raising the chances of effective therapy.

Early infection detection through these advanced wearable biosensors guarantees timely interventions, reduces the risk of complications, and supports personalized wound care strategies for significantly improved patient outcomes.

### 4.3. Therapeutic Feedback Systems

Therapeutic feedback systems essentially encompass the combination of biosensors and drug delivery mechanisms, therefore introducing a new phase in wound management [166]. One such example is that of a closed-loop, real-time monitoring of a biomarker for a wound, enabling immediate automated therapeutic intervention by delivering the medication at the right time [167]. The ability to incessantly monitor biomarkers—such as pH, cytokines, temperature, or enzyme activity—is essential to the systems. Deviations from normal levels, sensed by integrated biosensors, may signal infection, inflammation, or delayed healing. The real-time data then actuate release mechanisms that provide on-demand drug delivery in creating self-regulating systems that work with minimal human intervention possible. For instance, a biosensor that responds to an increased concentration of inflammatory cytokines, TNF-α, triggers the delivery of anti-inflammatory agents directly to the wound site.

A very good example of such technology includes microneedle-based systems. These devices use micro-scale needles capable of penetrating skin and hence delivering drugs accurately into the wound bed [168]. Coupled with biosensors, upon the detection of specific biomarkers, microneedles release antibiotics, anti-inflammatory drugs, or growth factors [169]. With this approach, the targeted delivery improves therapeutic efficacy but reduces systemic side effects.

**Figure 5:**
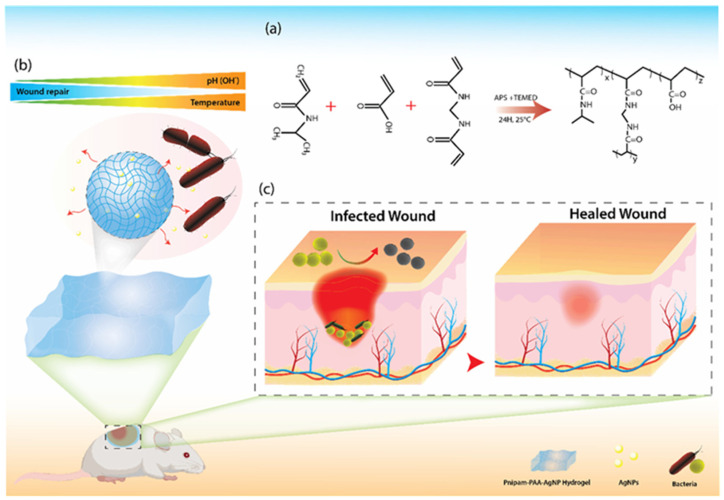
Bacteria-activated dual pH- and temperature-responsive hydrogel for infection control and wound healing. (**a**) Schematic of hydrogel cross-linked with *N*-isopropylacrylamide and acrylic acid, loaded with ultrasmall silver nanoparticles (AgNPs). (**b**) pH- and temperature-triggered Ag^+^ ion release, with restricted release at acidic pH (<5.5) and >90% release at alkaline pH (>7.4). (**c**) In vivo studies demonstrating clearance of Staphylococcus aureus infection and significantly accelerated wound healing. Copyright ACS Publications (2022) [171].

Another state-of-the-art design involves hydrogel-based drug delivery systems coupled with biosensors. Hydrogels can serve as a carrier matrix encapsulating therapeutic agents, which may be an antimicrobial peptide or bioactive molecules released in response to defined changes in pH values and/or the presence of an enzyme [170]. Thus, antibiotic release from a hydrogel dressing could be triggered by alkaline pH, a typical sign of infection, keeping the environment conducive for optimal wound healing simultaneously combating any infection [171] (Figure 5).

Another application of the therapeutic feedback systems is in the smart wound dressings embedded with sensor-drug delivery hybrids [172]. The smart wound dressing can sense and be capable of dispensing more than one kind of drug at a time, so that a dressing may notice high bacterial activity and start dispensing an antibiotic and an anti-inflammatory together to deal with infection and inflammation [173,174].

Thus integrated into the system, it acquires functionality in analyzing biomarker data and optimizes drug release patterns by incorporation with AI. From a different view, AI algorithms can learn from patient data regarding wound behavior prediction to change treatment strategies over the course of time for giving personalized care.

Therapeutic feedback systems are well placed to redefine wound management, offering a proactive, precise, and patient-centered approach to care. By automating the process of detection and treatment, these systems remove the dependency on frequent clinical interventions while improving outcomes of healing and significantly lowering the risk of complications.

## 5. Challenges and Limitations

### 5.1. Technical Issues

There are a number of technical obstacles that are associated with the development of wearable biosensors that are used for wound monitoring [175,176]. These issues include factors relating to accuracy, sensitivity, and durability [177]. Biomarkers of a wound, including pH, temperature, and inflammatory markers, can be measured with a high degree of precision thanks to their dependability. However, this would easily be compromised by sources of interference such as sensor drift or biofluids, whereas non-target molecules create the need for enhanced specificity through advanced materials and optimization of designs [60,178]. Another important aspect is that the biosensors should be sensitive enough to enable the detection of small biomarker concentrations that allow early-stage infection or subtle changes in wound conditions [179]. Such sensitivities are mostly realized with either complex sensor architectures or via enhancements by nanomaterials, thus adding more costs [180]. Other challenges are also applied to the durability of such biosensors since wearable biosensors should be able to perform well for extended use in dynamic and wet wound conditions [181]. Long-term exposure to biofluids, along with mechanical and environmental factors, may lead to a deterioration of sensor components; hence, this can lead to poor performance or failure. Due to the discussed limitations, above the current active areas of research in making new materials capable of self-healing, biofouling-resistant coatings, and robust encapsulation techniques, considering the said technical issues, have been viewed to be ongoing. In contrast, while much progress is achieved, this becomes a continuing impetus for more research and further development to make the device more reliable and pragmatic in real-world applications in wound care.

The lifespan of the operation of various sensing technologies used for wound monitoring is varied greatly. Electrochemical sensors can have a life from several days to one week, depending on electrode materials and enzyme-based coatings, but even biofouling and electrode degradation can compromise long-term performance. Optical sensors, especially fluorescent or plasmonic-based sensors, can have lifetimes of several weeks, but photo-bleaching and sensor drift can compromise accuracy over time. Colorimetric sensors are also reported to possess short useful lifetimes (24–72 h) owing to irreversible chemical reactions [182]. For the wound healing application, target sensor lifetimes would be similar to the wound healing process, which is typically 2–4 weeks for an acute wound and months for a chronic wound. To realize this, material breakthroughs such as self-sustaining enzyme coatings, anti-biofouling nanomaterials, and energy-aware sensor designs would be needed [183,184]. Further innovation is required to develop fully biodegradable, long-term stable, and self-powered biosensors that can continuously monitor wound biomarkers without frequent replacement.

### 5.2. Clinical Translation

Clinical translation of wearable biosensors for wound monitoring faces various challenges, especially those related to regulatory approval, patient compliance, and cost [185]. Biosensors are subject to stringent regulatory requirements by agencies such as the FDA and EMA concerning safety and efficacy; therefore, one of the greatest regulatory challenges is satisfying these requirements [186,187]. The regulatory path may be long and complex, with a large number of clinical trials and data required on biocompatibility, durability, and long-term performance. One big issue will be that of the high cost in both development and manufacturing. Wearable biosensors in particular can be so expensive, especially with advanced materials and technologies being integrated, that their accessibility becomes hard, mostly within low-resource settings and poor healthcare coverage for the patient [188]. Additionally, patient compliance is an issue in consideration, in that the device must feel comfortable and non-obstructive and have a friendly user interface during use. There might also be resistance to wearing sensors that cause irritation or discomfort or require repeated maintenance [189]. There is a need for education and training in using the devices properly, since improper application or lack of understanding can result in false results. Many of the challenges would require multi-stakeholder collaboration among researchers, healthcare providers, and regulatory bodies to streamline the approval processes, reduce the manufacturing costs, and come up with user-friendly systems that could enhance patient compliance.

Animal models like the murine excisional wound model are used universally for their prospects of human-like inflammatory and healing responses to enable sensor testing in measuring critical biomarkers such as pH, cytokines, and reactive oxygen species (ROS) [190,191,192]. Diabetic rat and porcine wound models are especially valuable for mimicking chronic wound conditions most similar to human diabetic ulcers and infection-prone wounds [193,194]. Additionally, details on in vitro phantom models have been incorporated, which afford controlled systems for the validation of sensors [195].

### 5.3. Power and Energy

Power and energy management is one of the big challenges in wearable biosensors, especially in the development of self-powered and battery-free systems [196,197,198]. These self-sustaining systems, which harvest energy from the ambient environment, including body heat, motion, or sweat, need to have adequate efficiency to allow continuous sensing without relying on any external energy source. All of these systems have energy density, efficiency, and reliability problems during long-term operation. For example, energy can be harvested from body movements or even sweat, which may not provide sufficient power to high-demand sensors—particularly those requiring real-time data processing and transmission—in a continuous manner [199]. Similarly, battery-less systems, while being beneficial in terms of eliminating the need for routine maintenance or disposal of batteries, face the problems of energy storage and transmission [200,201]. In many cases, small batteries or energy storage systems cannot supply adequate power for extended operating times without adding considerable weight or volume to the device at the risk of patient comfort and the effectiveness of the sensors [202]. Another crucial issue refers to the complexity of sensor power consumption management so that sensors can function independently without recharging frequently in a dynamic real-life environment [203]. The energy constraint imposes new requirements for wearables that could be responded to only through advances in low-power electronics, new energy-efficient materials, and novel ways of power harvesting for widespread adoption in clinical settings and consumer uses.

## 6. Future Perspectives

### 6.1. Emerging Technologies Such as Bioresorbable Sensors and Multi-Biomarker Platforms

**Figure 6:**
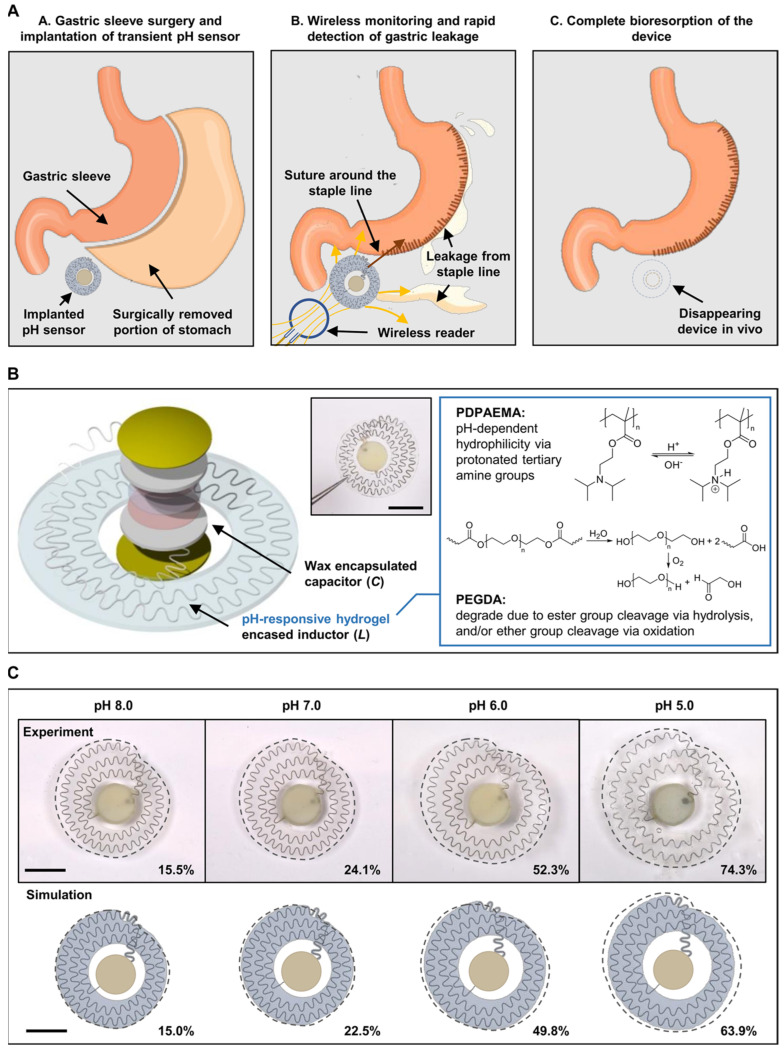
A bioresorbable pH sensor for wireless monitoring of pH. (**A**) Illustration for medical application of the proposed sensor in locally monitoring gastric leakage after LSG surgery. (**B**) Structure of the pH sensor and its compositions. (**C**) Experimental and simulation results of pH-triggered physical expansion of the sensor after 2 h of immersion in solutions of varying pH. Copyright AAAS (2024) [204].

Wearable biosensors have the potential to transform wound healing and infection monitoring through emerging technologies. Advancements in this field will be driven by bioresorbable sensors and multi-biomarker platforms [204,205,206]. These innovative methods are expected to substantially improve the capabilities of real-time monitoring and therapeutic interventions (Figure 6).

Bioresorbable sensors are one of the most crucial steps toward biocompatible, minimally invasive sensor technologies. They are designed in such a manner that, under natural conditions, their material degrades within the body over time and would not need removal or replacement [207,208]. Made from materials like polylactic acid (PLA), silk fibroin, or any other biodegradable polymer, bioresorbable sensors have a special edge over wound care [209,210]. They are directly implantable into the dressings or even embedded within the wound site, which allows for the monitoring of healing processes without any remaining foreign materials. As the healing process goes on, these materials deteriorate over time, lowering the chance of infection and the requirement for surgical removal. Examples include implantable bioresorbable pH sensors that detect bacterial activity and wound acidity levels [211]. These sensors release antimicrobials as necessary and dissolve without leaving any harmful residues after the wound has healed [212]. These devices offer increased comfort for the patient and a smooth experience, especially for chronic or large wounds where the monitoring period is long.

The multi-biomarker detection concept is being put forward here in order to provide enhanced multi-biomarker platforms for thorough wound management. These platforms integrate multiple sensors that simultaneously detect biomarkers, making the diagnostics of the wound environment exhaustive [213]. Multi-biomarker detection frameworks have been able to perform analysis of parameters involving inflammatory cytokines, pH levels, temperature, and bacterial metabolites in the wound by providing a complex overview [214]. The integration of various biomarkers enables these platforms to better detect infection, inflammation, and tissue degeneration, allowing for early intervention and tailored treatment. Such multi-biomarker systems could monitor wound healing progress and allow healthcare providers to change treatment strategies in real time [215]. For example, the combination of pH and temperature sensors might increase the ability to detect infections, while monitoring cytokines could give insight into the immune response of the wound [216].

On top of the integration with AI and IoT, these technologies bring predictive analytics and personalized care to the bedside, giving a much-needed impetus to the use of smarter and more effective wound management strategies. Further development in bioresorbable sensors and multi-biomarker platforms promises even better outcomes for patients with continuous, real-time insight through minimally invasive means. The development represents an exciting frontier in the field of wound care and brings leading-edge materials science together with sophisticated data analysis to develop more effective, efficient, and patient-friendly healthcare solutions.

### 6.2. Validation and Application of Wound-Healing Biosensors in Clinical Settings

Several novel biosensors have been clinically validated, showing their potential for practical wound healing and infection detection. A breakthrough in innovation is the PETAL sensor patch created by researchers at the National University of Singapore. The AI-powered, battery-free patch was clinically validated and achieved 97% accuracy in differentiating between healing and non-healing wounds through monitoring of the most important biomarkers such as pH, temperature, UA, and bacterial metabolites [50]. Sensor-assisted wound therapy, facilitated by implanted biosensors, significantly improved rates of healing for plantar diabetic foot ulcers without compromising patient mobility. The biosensors, which were implanted into offloading devices that were non-removable (felt soles, felt-fiberglass soles, or total contact casts with a ventral window), monitored crucial parameters such as pressure, temperature, humidity, and steps. Pressure sensing alarms, conveyed via smartwatch and internet apps, gave timely feedback to the patient, staff, and telemedicine units where pressure was higher than 125 kPa. In a multicenter randomized controlled clinical trial, an intervention group revealed faster healing (median 40.5 vs. 266 days) and lower rates of ulcer area expansions (7.9% vs. 29.7%) than controls, demonstrating the essential role of biosensors in achieving improved wound healing without impairing mobility [217]. These clinical examples capture how biosensors are being designed as effective, real-time, and personalized wound devices that provide quicker diagnosis, earlier infection detection, and better patient outcomes.

### 6.3. Potential of AI and Machine Learning for Predictive Analytics

Artificial intelligence and machine learning incorporated into wearable biosensor technologies hold great potential for a major improvement in predictive analytics for wound healing and infection monitoring. AI and ML analyze vast amounts of complex, real-time data from sensors and offer powerful insights that drive personalized care and early intervention—a huge paradigm shift in how wound management is approached [218].

Using those sensors, AI and ML algorithms can analyze data on pH levels, temperature, moisture, cytokine markers, and bacterial metabolites to provide insight into a wound’s potential trajectory and predict possible complications [219]. Those algorithms could learn from past data and recognize a pattern that may be very hard to detect by human clinicians. Similarly, a machine learning model trained on large datasets relating to the outcomes of wound healing could alert, based on small shifts in biomarker levels—say, minor elevations in pH or temperature—that a wound is at risk for infection. The earlier the detection, the sooner the course of infection can be altered, possibly reducing potential complications and speeding up the healing time.

Moreover, AI and ML have enabled predictive modeling; that is, these algorithms also project the future course that wound healing will take based on trends observed over time [219]. If data associated with the patient’s wound is regularly monitored and input into the AI system, it sends predictive information related to the wound condition to clinicians for needed adjustments in treatment. Similarly, AI models can suggest a certain type of dressing or application of medication at certain times of the wound’s state and expected healing curve. It creates a personalization of care to make the most effective treatment strategies tailored to the particular patient.

Integration of artificial intelligence and machine learning with Internet of Things-enabled biosensors opens up more possibilities in remote health monitoring and telemedicine applications [220]. Machine learning algorithms can analyze data in real time, therefore allowing health professionals to have real-time information about the status of a patient’s wound, even if remotely located. By increasing predictive capability, improved outcomes of wound care can occur, and at the same time, it empowers patients with insight into the healing process, thereby supporting self-management and decreasing frequency of clinical visits.

With more advanced development of AI and machine learning, predictive analytics will continue to make these practices even more effective, efficient, and timely in the provision of care. It is only going to further enhance both patient outcomes and a better level of healthcare experience through the next step of intelligent and data-driven medicine by prediction of complications, personalization of treatment plans, and automation of clinical decision making.

### 6.4. Opportunities for Large-Scale Adoption in Telemedicine and Personalized Care

Large-scale adoption of wearable biosensors for telemedicine and personalized care holds great potential, opening up new frontiers in the management of wound healing and infection monitoring [220]. With increasing remote healthcare and smart healthcare devices, patients can now access continuous, real-time monitoring and personalized treatment options from the comfort of their homes, thus greatly improving accessibility and efficiency in healthcare systems.

Wearable biosensors will add a new dimension to wound care, with telemedicine support, through the remote monitoring of wound status by healthcare providers and reducing frequent visits [111]. Biosensors capable of monitoring biomarkers such as pH, temperature, cytokine levels, and bacterial activity can wirelessly transmit real-time data to clinicians via cloud-based platforms. In general, this allows healthcare professionals to make timely decisions from continuous streams of data coming from a patient who can be far away from any healthcare facility. This increases efficiency in caregiving by reducing visits to hospitals in situations requiring recurring visits, such as the caregiving of chronic or difficult-to-cure wounds like diabetic ulcers or post-surgery wounds, and thus reduces overall healthcare costs while offering comfort to the patient [221]. Telemedicine can also democratize healthcare and improve outcomes by extending expert wound care to rural or otherwise underserved populations of patients.

Wearable biosensors also hold immense promise in the personalization of care. Such wearables may provide specific, patient-specific information that is useful in elaborating personalized treatment strategies for a particular wound. For instance, biosensors monitoring microenvironments around a wound—moisture, temperature fluctuations, and infection markers—inform the clinician of optimum times for dressing changes, topical treatments, or antibiotics [222]. This information might include the patient’s healing curve, which can be calculated using machine learning models operating on the data to suggest dynamic adjustments in treatment protocols. This kind of personalized, data-driven care is a lot more precise and thus can result in faster healing, fewer complications, and better patient outcomes.

This process is taken a step further by telemedicine platforms integrated with the use of AI, which may offer predictive insights into automating such decision making. AI learns from extensive data and thus can be designed to automatically flag those potential issues before they turn apparent to human eyes—infections or slow healing to enable timely interventions which would prevent worse complications from occurring [223].

In the near future, wearable biosensors combined with AI and telemedicine will offer an integrated, scalable solution for personalized wound care in improving both quality of care and patient satisfaction. Healthcare systems will be relieved of part of the strain as a result of these technologies, which will change wound care from reactive to proactive and involve patients in their own healing process.

## 7. Conclusions

Wearable biosensors are now set to revolutionize the face of wound care through continuous, accurate, real-time monitoring of critical wound parameters such as pH, temperature, moisture, and biomarkers of inflammation and infection. These provide unparalleled opportunities to improve patient outcomes with appropriate and timely clinical intervention. Wearable biosensors are being rendered more adaptive, reliable, and patient-friendly through advances in flexible and bioresorbable materials, multi-biomarker detection systems, and microfabrication techniques. Integration with artificial intelligence and machine learning provides additional sophistication to predictive analysis, enabling healing trajectory analysis and personalized treatment regimens. These smart systems work not only on optimizing therapeutic response but also bringing actionable insights for healthcare professionals to prevent complications and readmissions to the hospital. In addition to that, wearables aligned with telemedicine route new options for remote and scalable solutions in wound management, which are very useful in response to disparities in health access. Continuous monitoring with minimal burden from frequent clinical visits may be especially beneficial to rural or medically underserved individuals. However, in spite of all these advances, there are many challenges yet to be overcome before wearable biosensors can be translated into wide clinical use. Regulatory issues, cost-effectiveness, energy-related issues, and patient compliance are some of the challenges that require serious research and collaboration across disciplines. Overcoming these barriers will be critical to the translation of these promising technologies into routine clinical practice. Wearable biosensors will no doubt become increasingly integral in the field of precision medicine, both in wound care and more generally in health monitoring, as this field continues to evolve. Their power to translate real-time data into actionable insight opens a new frontier for proactive, efficient, patient-centered care. Such systems will overcome existing limitations and foster innovation, thus setting new standards in wound management and improving healthcare outcomes worldwide.

## Figures and Tables

**Table 1:** Biomarkers and their clinically relevant ranges for wound healing and infection monitoring.

Biomarker	Normal Range	Clinically Relevant Range	Detection Sensitivity	Relevance in Wound Healing	Measurement Location	Reference
Temperature	31.1–36.5 °C	≥3 °C above surrounding skin	±0.5 °C	Indicates localized infection and inflammation	Wound Fluid, Capillary Blood	[110,111,112]
pH	4.2–5.6	≥7 (7.15–8.9 in chronic wounds)	±0.1 pH units	Helps differentiate chronic wounds from healing wounds, indicating infection or delayed healing	Wound Fluid, Interstitial Fluid	[34,111,112]
Uric acid	220–750 µM	>1 mM (chronic wounds leg ulcers)	µM range	Elevated levels suggest oxidative stress, which can delay wound healing.	Wound Fluid, Interstitial Fluid	[112,113]
Interleukin-1 (IL-1)	<5 pg/mL	≥5 pg/mL	pg/mL range	A key inflammatory biomarker, elevated in infected wounds	Capillary Blood, Wound Fluid	[114,115]
Interleukin-6 (IL-6)	<2.4 pg/mL	≥2.4 pg/mL	pg/mL range	A key inflammatory biomarker, elevated in infected wounds	Capillary Blood, Wound Fluid	[114,115]
Tumor Necrosis Factor-alpha (TNF-α)	<14 pg/mL	≥14 pg/mL	pg/mL range	Elevated levels are associated with chronic inflammation, indicating infection or delayed healing.	Capillary Blood	[115]
Trimethylamine (TMA)	Not typically present	>30 (ppm) Elevated in infected wounds	ppm	Marker for bacterial activity, particularly in anaerobic infections	Wound Fluid	[50]

## Data Availability

Not applicable.
